# Structural basis of connexin-36 gap junction channel inhibition

**DOI:** 10.1038/s41421-024-00691-y

**Published:** 2024-06-18

**Authors:** Xinyue Ding, Simone Aureli, Anand Vaithia, Pia Lavriha, Dina Schuster, Basavraj Khanppnavar, Xiaodan Li, Thorsten B. Blum, Paola Picotti, Francesco L. Gervasio, Volodymyr M. Korkhov

**Affiliations:** 1 Laboratory of Biomolecular Research, Paul Scherrer Institute, Villigen, Switzerland; 2 School of Pharmaceutical Sciences, University of Geneva, Geneva, Switzerland; 3 ISPSO, University of Geneva, Geneva, Switzerland; 4 Swiss Institute of Bioinformatics, University of Geneve, Geneva, Switzerland; 5 Institute of Molecular Systems Biology, ETH Zurich, Zurich, Switzerland; 6 Institute of Molecular Biology and Biophysics, ETH Zurich, Zurich, Switzerland; 7 Department of Chemistry, University College London, London, UK

**Keywords:** Cryoelectron microscopy, Ion channel signalling

Dear Editor,

Connexins (Cxs) assemble as hexameric hemichannels (HCs) that reach the cellular surface and dock with their counterparts on the neighboring cells, forming the gap junction channels (GJCs) and coupling cells metabolically and electrically. Pancreatic Cx36 plays a crucial role in insulin secretion^1^, whereas in the brain Cx36 is involved in neuronal synchronization^2^. Dysregulation of Cx36 is associated with epilepsy, traumatic brain injury, and ischemia^3^, and elevated gap junction coupling in these conditions contributes to neuronal death. The development of selective inhibitors of Cx36 could be of therapeutic value. Although a structure of Cx36 has been determined recently^4^, it remains unclear how drugs inhibit the Cx36 and other Cx channels.

Three antimalarial drugs, mefloquine, quinine, and quinidine (Supplementary Figs. S1a, S2), specifically inhibit the Cx36 channel^5,6^. As antimalarial drugs, mefloquine and quinine target *P. falciparum* purine nucleoside phosphorylase^7^, and mefloquine may also target *Plasmodium falciparum* 80S ribosome^8^. However, mefloquine also causes severe cardiac, neurological, and psychiatric side effects^9^. Quinine causes cardiovascular side effects, blood disorders, and cinchonism^10^, while quinidine serves both as an antimalarial and an anti-arrhythmic drug^11^, and may cause cinchonism. The molecular mechanisms underlying these three drugs’ side effects are unknown but likely involve disruption of normal Cx36 coupling. The neuropsychiatric adverse effects associated with mefloquine align with the expected consequences of Cx36 dysregulation. Quinine and mefloquine selectively act on Cx36 and Cx50, suggesting Cx inhibition as a potential mechanism for the drug side effects^5^. Mefloquine-induced inhibition of Cx36 may contribute to region-specific neuronal hyperactivity and increased susceptibility to epileptic events^12^. Additionally, mefloquine, quinine, and quinidine inhibit spreading depolarization episodes, which requires gap junction coupling^13^. We set out to characterize the structure of Cx36 in the absence and presence of mefloquine, quinine, and quinidine, and to determine the general principles of Cx channel inhibition by small molecules.

We purified the human Cx36 (Supplementary Fig. S1b–e) and performed ligand binding assays (Supplementary Fig. S2a–e), revealing micromolar affinities of the three drugs. Cx36 with and without the drugs (1 mM) was analyzed by cryo-EM, yielding four reconstructions (Fig. 1a, b; Supplementary Figs. S3–S7 and Table S1): Cx36–mefloquine (Cx36–mfq), Cx36–quinine (Cx36–quin), Cx36–quinidine (Cx36–quid) and apo-Cx36, at 2.14 Å, 2.73 Å, 2.9 Å, and 2.49 Å resolution, respectively. The overall protein conformations are nearly identical (Fig. 1a, b; Supplementary Fig. S7), similar to the recently published Cx36 structure^4^. The N-terminal gating helix (NTH) is unresolved in our reconstructions (Supplementary Fig. S7b). It is possible that in the detergent environment, the NTH is too dynamic to be captured by cryo-EM. The major differences were found in the pore region of the channel: Cx36–mfq, Cx36–quin, and Cx36–quid feature additional densities, corresponding to six bound drugs per Cx36 hexamer (Fig. 1b–d; Supplementary Fig. S7d).

**Fig. 1:**
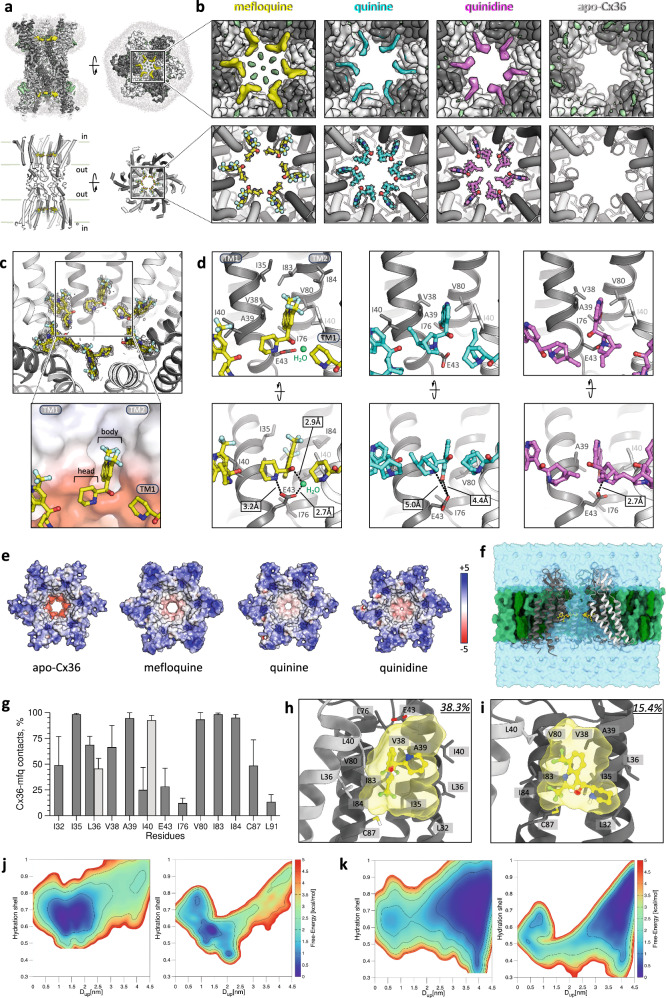
Structural basis of Cx36 GJC inhibition by antimalarial drugs. **a** Cryo-EM map and model of mefloquine-bound Cx36. Yellow density (top) — bound mefloquine; individual Cx36 monomers are colored white and gray; light gray density — detergent micelles. **b** Zoomed-in views of maps (top) and models (bottom) of the mefloquine-, quinine- and quinidine-bound Cx36 (yellow, cyan, magenta, respectively), compared to apo-Cx36 (right-most); all maps contoured at 5σ. **c** Densities of 6 mefloquine molecules (mesh, post-processed density map, 3σ). Inset: the planar mefloquine “body” is inserted into a hydrophobic groove between TM1 and TM2 of one Cx36 monomer and a portion of TM1 of the neighboring monomer; the head-group (“head”) orients towards the pore, making contacts with the polar region of the TM1 and with the neighboring mefloquine molecule. **d** A detailed representation of the binding site residues. **e** The drug-free (apo-Cx36) and drug-bound structures of Cx36, in surface representation, colored according to electrostatic potential (scale bar: –5/+5 kT/e). Drug binding changes pore electrostatics (mefloquine) and/or introduces a steric barrier (quinine, quinidine). **f** Cross-section of 6mfq–Cx36 HC. Individual Cx36 monomers are colored light and dark gray; mefloquine — yellow, POPC — light green, cholesterol — dark green, water — transparent cyan. **g** Average frequencies of occurrence of the contacts between Cx36 and mefloquine in the 6mfq–Cx36 MD simulations. Residues in different monomers are colored light and dark gray. Error bars represent the standard deviation. **h**, **i** Interactions established by mefloquine in cluster families C1 (**h**) and C2 (**i**). Their own frequency of occurrence during the 6mfq–Cx36 MD simulation is reported in the top right corners. The solvent-accessible surface of mefloquine is colored transparent yellow. **j**, **k** Free-energy surfaces associated with K^+^ (**j**) and Cl^–^ (**k**) permeation across the Cx36 hexamer in the apo-Cx36 (left) and 6mfq–Cx36 (right) systems. The maps are colored according to the right sidebars while isolines are placed every 1 kcal/mol.

The structures reveal three key elements of the drugs relevant to Cx channel interactions: (i) a planar group that inserts into the pocket formed by residues in the transmembrane1 (TM1) and TM2 (the “body”); (ii) a hydrophobic “head-group” extending into the pore and contacting the neighboring drug molecules; (iii) a head-group nitrogen atom contacting the conserved negatively charged residue at the pocket (E43) (Fig. 1c, d). The high-resolution Cx36–mfq 3D reconstruction gives the greatest insight into the atomic details of inhibitor binding. The body of the drug wedges itself into the pocket at the interface of two Cx36 monomers, interacting with the residues I35, V38, A39, and I40 (and the neighboring subunit I40) in TM1, and I76, V80, I83, and I84 in TM2 (Fig. 1c). The pocket is hydrophobic, and multiple weak non-polar interactions and geometric complementarity between mefloquine and the pocket likely drive this interaction. The piperidinium head-group of mefloquine is asymmetrically extending toward the pore. The binding pose of mefloquine is stereoselective: although we used a racemic mixture of the drug, the high-resolution structure captured the S/R-enantiomer. E43 directly coordinates the N atom of the piperidinyl head-group, and links to the nearby hydroxyl via an ordered water molecule (Fig. 1d, left; Supplementary Fig. S6e). The drug-binding site residues are relatively poorly conserved, despite the structural conservation of this pocket (Supplementary Figs. S8, S9, and Supplementary Discussion).

In the case of the quinine- and quinidine-bound Cx36, the drugs are somewhat less well resolved at the nominal resolution of 2.73 Å and 2.9 Å, respectively (compared to mefloquine in the 2.14 Å cryo-EM map). This disparity in the drug density may be attributed to the lower *K*_d_ of quinine/quinidine binding to Cx36 compared to mefloquine. Nevertheless, the density map quality allows us to model the drugs based on the observed features confidently. In both cases, the body of the drug engages in fewer hydrophobic contacts, and a very distinct head-group points towards the pore and makes contacts with the neighboring ligand head-group and with the E43 residue (Fig. 1b, d, middle & right; Supplementary Fig. S6f, g). Although quinine and quinidine are stereoisomers, each is readily accommodated within its Cx36-binding site. Thus, the binding of each of the three compounds creates two hydrophobic rings in the translocation pathway in the GJC (Fig. 1e). Moreover, in the case of quinidine, the drug in the observed conformation completely closes the pore.

To investigate the dynamic behavior of the mefloquine-bound Cx36 complex and its impact on ion permeation, we performed molecular dynamics (MD) simulations with apo- and mefloquine-bound Cx36 (300 ns-long MD simulations of Cx36 HC, apo-Cx36, and Cx36–6mfq). The presence of six ligands minimally affected Cx36 conformational flexibility, with mefloquine primarily establishing hydrophobic interactions with adjacent residues, displaying two main conformations, one resembling the cryo-EM structure and the other losing specific interactions (Fig. 1f–i; Supplementary Fig. S10). Additionally, we identified phospholipid binding sites on the HC’s surface, notably finding the oleic acid chain of POPC targeting a hydrophobic cavity between the adjacent Cx36 monomers and the palmitic acid chain interacting with the P247–L275 helix (Supplementary Fig. 11). Further details on ion translocation in apo-Cx36 and Cx36–6mfq MD simulations can be found in Supplementary Table S2.

To evaluate the impact of mefloquine on ion permeation through Cx36, we employed a collective-variable (CV)-based free energy algorithm, On-the-fly Probability Enhanced Sampling (OPES). This method enables the sampling of ion crossings and associated free energy profiles by swiftly constructing a bias potential through on-the-fly probability estimation along selected CVs. We performed > 200 ns OPES simulations on the apo-Cx36 and Cx36–6mfq systems and sampled ion permeation by employing CV “D_up_”, i.e., the distance of K^+^/Cl^–^ concerning the extracellular side of the HC (apo-Cx36_K^+^, apo-Cx36_Cl^–^, 6mfq–Cx36_K^+^ and 6mfq–Cx36_Cl^–^; Supplementary Fig. S10f). As shown in Supplementary Fig. S10g, the free-energy profile of K^+^ in apo-Cx36_K^*+*^ and 6mfq–Cx36_K^+^ strongly differs at the transition state (D_up_~3.0 nm), where the mefloquine ligands are located. Conversely, the free energy of the two main basins (D_up _~ 1.5 nm and D_up_ ~ 4.0 nm) appears unaffected by mefloquine ligands, as indicated by a consistent difference of ~2.0 kcal/mol measured over the OPES simulation time (see Supplementary Fig. S10i). A similar scenario can also be observed for the permeation-free energy of Cl^–^, whose profile differs around the value of D_up_ ~ 1.5–3.0 nm between apo-Cx36_Cl^–^ and 6mfq–Cx36_Cl^–^ (Supplementary Fig. S10h). Instead, the two basins at D_up_ ~ 1.0 nm and D_up_ ~ 4.0 nm are almost identical in value, and their difference along the computational time is measured to be ~–1.7 kcal/mol (Supplementary Fig. S10j).

The free energy surfaces reconstructed by OPES indicate that mefloquine inhibits Cx36 by altering ion permeation kinetics rather than thermodynamics. This could result from a pore size reduction upon the drug, compelling ions to shed more than 50% of their hydration shells to traverse the pore (Fig. 1j, k). Curiously, the Cl^–^ ions exhibited different hydration values in the intracellular side of Cx36’s cavity, ranging from “fully hydrated” (~0.9) to “poorly hydrated” (0.4) (scale: 0–1), possibly due to the presence of a pronounced positive electrostatic potential on the intracellular side of Cx36, inducing Cl^–^-protein surface interactions (Supplementary Fig. S10j). Thus, although a complete channel blockage does not occur, the ion flux is substantially reduced upon mefloquine binding.

In conclusion, the drug–Cx interaction relies not only on the complementarity between the drug and the protein pocket, but also on the geometry and physical properties of the drug itself (further discussion can be found in Supplementary Discussion). The neighboring drug molecules within the pore interact with each other, forming a hydrophobic ring within the pore that either obstructs the pore completely or introduces the electrostatic barrier that limits solute translocation through the channel (Supplementary Fig. S12). This mode of Cx inhibition by the small molecules is unique and may present an attractive new approach to Cx drug discovery.

### Supplementary information

Supplementary Information

## Data Availability

The atomic coordinates and the density maps have been deposited in the Protein Data Bank and Electron Microscopy Data Bank, respectively, with the following accession numbers: PDB ID 8QOJ, EMD-18540; PDB ID 8R7P, EMD-18987; PDB ID 8R7Q, EMD-18988; PDB ID 8R7R, EMD-18989. All other data are available in the main text or the supplementary materials.
