# Supplementary material for: Structural basis of connexin-36 gap junction channel inhibition
Source: Cell Discov. 2024 Jun 18;10:68. doi: 10.1038/s41421-024-00691-y (PMC11189382; doi:10.1038/s41421-024-00691-y)
Supplement: Supplementary file 1 — Supplementary Information [file 41421_2024_691_MOESM1_ESM.pdf]

Supplementary Information

Title: **Structural basis of connexin-36 gap junction channel inhibition**

Xinyue Ding[1,†], Simone Aureli[2,3,4,†], Anand Vaithia[1], Pia Lavriha[1], Dina Schuster[5,6], Basavraj Khanppnavar[1], Xiaodan Li[1], Thorsten B. Blum[1], Paola Picotti[6], Francesco L. Gervasio[2,3,4,7*], Volodymyr M. Korkhov[1,6,*]

[1] Laboratory of Biomolecular Research, Paul Scherrer Institute, Villigen, Switzerland

[2] School of Pharmaceutical Sciences, University of Geneva, CH-1211 Geneva, Switzerland

[3] ISPSO, University of Geneva, CH-1211 Geneva, Switzerland

[4] Swiss Institute of Bioinformatics, University of Geneve, Geneva, CH-1206, Switzerland

[5] Institute of Molecular Systems Biology, ETH Zurich, Switzerland

[6] Institute of Molecular Biology and Biophysics, ETH Zurich, Switzerland

[7] Department of Chemistry, University College London, WC1E 6BT London, UK

† These authors contributed equally

* Corresponding authors:

Francesco.Gervasio@unige.ch and volodymyr.korkhov@psi.ch

This pdf file includes:

Materials and Methods

Supplementary Discussion

Supplementary Figs. 1-12

Supplementary Tables 1-2

Supplementary References (1-42)

Materials and Methods

Connexin-36 Expression

A synthetic gene encoding human connexin 36 (Cx36, UniprotID Q9UKL4; synthesis performed by Genewiz) with a C-terminal 3C-EYFP-twinStrep tag was cloned into the pACMV vector (REF). Freestyle 293 cells (HEK293F) cells were grown at 37°C in Dulbecco's Modified Eagle's medium (DMEM) supplemented with 10% fetal bovine serum (FBS) and then exchanged to DMEM supplemented with 2% FBS prior to transfection. The cells were transiently transfected with the expression plasmid using branched polyethyleneimine (PEI; Sigma Aldrich) at a ratio of 1:2 (w/w; DNA:PEI). After 48 hours of incubation at 37°C, cells were harvested using a cell scraper, frozen, and stored at -80°C until further use.

Connexin-36 Purification

Frozen cells were thawed and resuspended in buffer A (25 mM Tris-HCl, pH 8.0, 150 mM NaCl) supplemented with protease inhibitor cocktail (04693132001; Roche, Basel, Switzerland). Cells were disrupted using a Vibra-Cell sonicator, employing a 0.5-second pulse per plate, separated by a 0.5-second pause, and operated at a 35% amplitude. After sonication, the membrane fraction was clarified by ultracentrifugation (Beckman Coulter Ti45 rotor, 35,000 rpm, 50 min) and solubilized in buffer A containing 1% dodecyl-β-D-maltopyranoside (DDM) and 0.2% cholesteryl hemisuccinate (CHS) by rotating at 4°C for 1 hour. Insoluble material was removed by another round of ultracentrifugation, and the supernatant was mixed with CNBr-activated Sepharose coupled with anti-GFP nanobody and incubated at 4°C for 30 min. The resin was collected using a gravity column and washed with at least 40 column volumes of buffer B (25 mM Tris-HCl, pH 8.0, 150 mM NaCl, 0.02% glyco-diosgenin (GDN)). Cx36 was eluted overnight by addition of HRV 3C protease (0.5 mg) at 4°C and then concentrated with a 100-kDa molecular weight cutoff concentrator. Protein was injected onto a Superose 6 Increase 10/300 GL column equilibrated with buffer B for further purification. The fractions corresponding to Cx36 were collected, concentrated, and used immediately for cryo-EM grid preparation.

Binding assays

The binding affinity of Cx36 for ligands was assessed using intrinsic tryptophan quenching. Briefly, 5 μM of purified protein was prepared in a solution containing 25 mM Tris-HCl (pH 8.0), 150 mM NaCl, and 0.02% GDN. The protein solution was titrated with increasing amounts of ligands at sixteen titration points, ranging from 0 μM to 92 μM. Each titration point was measured using a Cary Eclipse spectrophotometer, exciting at 295 nm and recording the emission in the range of 300-500 nm. For the control titration of quinine and quinidine, drug alone in buffer was used to obtain the baseline fluorescence, which was then subtracted from the fluorescence of Cx36 in the presence of respective drug. The relative fluorescent intensity at 325 nm was plotted against the concentration of the drugs to generate the quenching profile. The apparent dissociation constant (K_d) values were determined by fitting the data to the specific binding with variable Hill slope model using GraphPad Prism 8.3.1.

Differential Scanning Fluorimetry (nano-DSF) analysis of Connexin36

A serial titration of mefloquine was mixed with an equal volume of 0.4 mg/ml Cx36 protein in a solution containing 25 mM Tris-HCl (pH 8.0), 150 mM NaCl, and 0.02% GDN. The mixture was loaded into Prometheus NT.48 Series nanoDSF Grade High Sensitivity capillaries (Nanotemper) and subjected to NanoDSF (thermal unfolding) using Prometheus Panta (Nanotemper). The temperature was continuously increased at a rate of 1°C/min from 15°C to 95°C. The raw data were analyzed with Panta Analysis software (Nanotemper) to obtain the first derivative of 350 nm/330 nm with respect to the temperature. The unfolding transition temperature (T_m) was determined as the maximum value of the

first derivative for each titration. The obtained T_m values were then plotted against mefloquine concentration and fitted with a one-site binding model using GraphPad Prism 8.3.1 software.

Cryo-EM analysis

Sample preparation and data collection. The purified Cx36 was concentrated to ~2.0 mg/mL. For the Cx36-mefloquine and Cx36-quinidine complex, a mefloquine or quinidine stock in DMSO was added to the protein to a final concentration of 1 mM. For the Cx36-quinine complex, a final concentration of 300 µM quinine in DMSO was used (at higher concentrations of the quinine the quality of the cryo-EM sample preparation deteriorated, manifesting in strongly aggregated particles judged by cryo-EM imaging as described below). The protein-drug mixture was incubated on ice for 30 min. The Quantifoil R1.2/1.3 200-mesh grids were glow-discharged for 30 s at 25 mA using a PELCO easiGlowTM glow-discharged system. A 3 µL aliquot of the protein was applied to the grid, which was then blotted and plunge-frozen in liquid ethane using a Vitrobot Mark IV (Thermo Fisher Scientific). The grids were stored in liquid nitrogen until the day of data collection. Micrographs were collected on a Titan Krios electron microscope equipped with a K3 direct electron detector and a GIF-Quantum energy filter for Cx36-mefloquine, Cx36-quinine, Cx36-quinidine and a GIF- BioContinuum energy filter for apo-Cx36 at ScopeM, ETH Zurich. The images were recorded using EPU2.0 software and dose-fractioned to 40 frames in super-resolution mode. The total dose per movie was 50, 55, 50, 55 e-/Å^2 for apo-Cx36, Cx36-mfq, Cx36-quin and Cx36-quid datasets, respectively.

Cryo-EM data processing. The micrographs were assigned into different optics groups according to the EPU beam shift values using a script developed by Dr. Pavel Afanasyev (ETH Zurich; https://github.com/afanasyevp/cryoem_tools). The movies were corrected using MotionCor2 [1] [1]nd Gctf[2] was used for CTF estimation. For apo-Cx36, 795 particles were manually picked within Relion 4.0 [3,4]. These particles then underwent 2D classification, revealing pronounced features consistent with gap junction channels (GJCs). The most discernible 2D classes were chosen to serve as templates for the automated particle picking process across all micrographs. For the later datasets of Cx36 with drugs, the refined map of apo-Cx36 was used as template for autopicking particles. After several rounds of 2D classifications, good 2D classes were selected and extracted for 3D classification and further 3D refinement with imposed D6 symmetry. To achieve enhanced resolution, CTF refinement and Bayesian polishing were performed using Relion4.0. Additionally, the pixel size was corrected to 0.65 Å or 0.66 Å, depending on the context: apo-Cx36 or Cx36-mefloquine, Cx36-quinine and Cx36-quinidine during the postprocessing step. Local resolution maps were calculated using ResMap [5] implemented in Relion 4.0. The detailed steps of image processing are shown in Supplementary Fig. S3-5 and Supplementary Table S1.

Model building, refinement and validation. The structure of apo-Cx36 was manually built in COOT [6]. The SWISS-MODEL homology model based on connexin-50 GJC (PDB ID 7JJP) of Cx36 was used as a guide for the apo-Cx36 structure. The apo-Cx36 was then used as a template for building the Cx36-mfq, Cx36-quin, Cx36-quid complexes. The cytoplasmic regions of Cx36 (M1-H18, K103-E193, A283-V321) were not built due to the poor quality of the corresponding regions in the density maps. A racemic mixture of mefloquine was added to the Cx36 protein, but only the (+)-mefloquine enantiomer (chemical ID YMZ) was found to bind to the pore of Cx36 according to the refined density map. Quinine (chemical ID QI9) from PDB 4UIL and quinidine (chemical ID QDN) from PDB 4WNU were fit into the density maps using rigid body fit. ALL the structures were refined using phenix.real_space_refine in PHENIX [7]. Model validation was performed as described previously [8]. Briefly, in order to generate FSC curve of model versus map, the coordinates of the final refined model was randomly modified 0.5 Å withing the PDB tool in Phenix. This perturbed model was subsequently subjected to refinement using one of the two available half maps. The refined model was then further iteratively refined using the other half map. The geometries of the models were validated using MolProbity [9]. All figures were prepared in PyMOL [10], Chimera [11] and ChimeraX [12].

Electrostatic surface potential calculations. The molecules underwent preparation for electrostatic calculations utilizing PDB2PQR [13] with the AMBER ff99 force field [14]. Determination of electrostatic surface potentials for the protein in the presence of ligands was performed using APBS Tools 2.1 [15] within PyMOL, utilizing the nonlinear Poisson-Boltzmann Equation.

Sample preparation for LC-MS/MS analysis

Purified protein (20 µg) was digested using a ProtiFi S-Trap™ micro spin column according to the manufacturer's protocol. The peptides were dried in a vacuum centrifuge and resuspended in 1 mL 5% acetonitrile (ACN), 0.1% formic acid (FA).

LC-MS/MS data acquisition

The protein samples (1 µL) were injected on a nano-flow LC system (Easy-nLC 1200, Thermo Fisher Scientific). Peptides were separated on a 40 cm x 0.75 µm (inner diameter) column packed in-house with 3 µm C18 beads at a flow-rate of 300 nL/min, a 60 min linear gradient from 3-30% II (Eluent I: 0.1% FA, Eluent II: 95% ACN, 0.1% FA) at 50°C. The samples was analyzed on an Orbitrap Exploris 480 mass spectrometer (Thermo Fisher Scientific). The samples was measured with a data-independent acquisition (DIA) method with 41 variable width DIA windows with a 1 m/z overlap. Survey MS1 spectra were recorded with a mass range between 350-1150 m/z at a resolution of 120,000 with 200% normalized AGC target or 264 ms maximum injection time. MS2 spectra covered a mass range of 150-1150 m/z at a resolution of 30,000. HCD collision energy for was set to 30% with 200% normalized AGC target or 66 ms maximum injection time.

LC-MS/MS Data analysis

Peptide identification and protein inference of DIA measurements was performed using Spectronaut™ software (Biognosys, version 15.5) in directDIA™ mode. Default settings were applied with minor adjustments. The minimal peptide length was set to 5 amino acids and single hits were excluded. The data were exported from Spectronaut and plots were prepared with GraphPad Prism 9.2.0. The raw file, as well as all relevant data analysis files have been deposited to the ProteomeXchange Consortium via the PRIDE [16] partner repository with the dataset identifier PXD044909.

Molecular dynamics simulations

Ligand parametrization. Mefloquine was parametrized using Antechamber with the general Amber force field 2 (GAFF2) [17] and RESP charges fitted to ab-initio calculations with Gaussian16 following standard procedures.

Hexamer MD simulations. The Cx36-mfq cryo-EM structure was used as the starting 3D structure. For each monomer, the cysteine couples *C55-C242*, *C62-C236*, and *C66-C231* have been bound with a disulfide bridge. This allowed us to obtain the following two systems: (i) *apo-Cx36*, and (ii) *6mfq-Cx36* (i.e., six Mefloquine bound inside the Connexin pore). The complexes thereby obtained were embedded into a tailored phospholipid bilayer using CHARMM-GUI [18] and solvated with TIP4P water model (salinity of 150 mM KCl). The N-terminus and C-terminus of each Connexin monomer were capped with an acetyl and a methyl-amino protecting groups, respectively. The DES-Amber force field was employed [19] in the MD engine GROMACS 2021.5 [20]. Each simulation box underwent a thermalization cycle using decreasing time-dependent restraints on heavy atoms with the following protocol: 1 ns of NVT simulation followed by 1 ns of NPT simulation for each temperature, starting from 100 K until 300 K with steps of 50 K. During the thermalization, the "V-rescale" thermostat has been employed, whereas, during the production run, we resorted to the Langevin dynamics temperature control scheme. The particle-mesh-Ewald (PME) method was used to treat the electrostatic interaction [21]. On the van

der Waals interactions, a cut-off distance of 1.0 nm was applied. The pressure was fixed at a reference value equal to 1 bar thanks to the "*C-rescale*" barostat [22].

Lipids analysis. By comparing the position of the lipids in the MD simulations with cryo-EM densities, we pinpointed phospholipid binding hotspots on the hemichannel's surface (see Supplementary Fig. 11a). Specifically, we found that the oleic acid chain of POPC exhibits an affinity for a hydrophobic cavity formed by two adjacent Cx36 monomers (see Supplementary Fig. 11b), while the palmitic acid chain interacts with the P247-L275 helix (see Supplementary Fig. 11c). Supplementary Fig. 11d-e depicts POPC binding to these external hydrophobic hotspot in MD simulations, improving fit to Cryo-EM densities as they reach their final pose.

OPES MD simulation. The passage of K^+ and Cl^- ions was investigated through enhanced sampling simulation, by employing the "On-the-fly probability enhanced sampling" algorithm [23]. Two different collective variables (CVs) were used to estimate the ions' translation free-energy. To discriminate between different location of Connexin's channel, three dummy atoms have been defined along the pore, i.e., "P_{up}", "P_{middle}", and "P_{down}" (see Supplementary Fig. S9f). For the sake of clarity, we defined P_{up} as the geometric center among T51's and M52's Cα of the six Cx36's monomers, P_{middle} as the geometric center among W79's Cα of the six Cx36's monomers, and P_{down} as the geometric center among T20's and M21's Cα of the six Cx36's monomers.

To enhance the sampling of the ions, we selected the "Distance" CV, monitoring the distance between the K^+ and Cl^- ions and the dummy atom P_{up} (i.e., "D_{up}"). To avoid unphysical "jump" of the ions across the periodic boundary conditions, a harmonic restraint was placed on their distance with respect to the dummy atom P_{middle}. A gaussian potential was applied on D_{up}, with an initial value of 30 kJ/mol and a deposition rate (i.e., pace) of 500 integration steps. To run the OPES simulation, the MD engine GROMACS 2021.5 patched with PLUMED 2.7.1 was employed. Regarding the thermostat and the barostat, we used the same protocol of the unbiased MD simulations. The OPES simulations to study the passage of K^+ and Cl^- ions have been replicated thrice for both *apo-Cx36* and *6mfq-Cx36* systems and carried out until convergence. To discriminate between different position inside the Cx36's pore, we also monitored the "*Hydration shell*" of the K^+ and Cl^-, an auxiliary CV employed to monitor the water coordination of the ions upon which we reweighted the collected bias potential. For additional information about the water coordination please refer to Refs [24,25].

Binding interface evaluation. To properly assess the interactions established by the residues of Cx36 and Mefloquine, the contacts between the hexamer and the ligands have been displayed as histograms, by counting their frequency of frequency of occurrence through the PLOT NA routine of "Drug Discovery Tool" (DDT) [26]. We defined a neighboring cutoff value of 4 Å between two interacting residues.

Cluster analysis. Cluster analyses on the MD trajectories were performed using GROMACS's gmx cluster routine, using the gromos algorithm. The cluster families of Cx36 in the apo-Cx36 and 6mfq-Cx36 MD simulations were obtained by aligning the trajectory on the Cα atoms of Cx36's secondary structure elements and computing the RMSD among the same sample of atoms. The cluster families on Mefloquine in the 6mfq-Cx36 MD simulation were obtained by aligning the trajectory on the Cα atoms of Cx36's secondary structure elements and computing the RMSD on Mefloquine's heavy atoms. The RMSD threshold value of 1.5 Å was chosen considering the number of cluster families generated and the similarity of protein conformations within a cluster family. The presence of six ligands had a negligible impact on the overall Cx36 conformational plasticity (RMSD ~1.0 Å, Supplementary Fig. S10a; low RMSF and RMSD-based cluster analysis, Supplementary Fig. S10b-d).

Cryo-EM rigid-body fitting. To investigate the positioning of the POPC lipid chains within the Cryo-EM map, we applied a rigid-body fitting procedure on the apo-Cx36 and 6mfq-Cx36 MD simulations. Specifically, we employed the stand-alone version of "Powerfit" [27]. The rotational sampling density was set at 5 degrees to ensure comprehensive exploration of conformational space.

Supplementary Discussion

Our structures reveal that the three drugs (mefloquine, quinine and quinidine) exhibit hydrophobic interactions with the neighboring molecules and engage with the conserved negatively charged residue at the pocket (E43). However, the distances of these interactions vary notably, with implications for their binding affinities and inhibitory potencies. The "body" groups of the three drugs all engage in hydrophobic contacts with the pore (**Fig. 1d**, Supplementary Fig. S6e-g). Mefloquine makes the most extensive contacts with six residues, attributed to the presence of two trifluoromethyl groups, which likely contribute to its heightened binding potency compared to quinine (four residues) and quinidine (two residues). Furthermore, the methoxy group of quinine and quinidine resides in a dehydrated hydrophobic environment, with the oxygen atom devoid of a potential hydrogen bond donor. This absence of one or two potential hydrogen bonds upon binding might explain the lower binding affinity of quinine and quinidine (Supplementary Fig. S6e-g).

Similarly to Cx36, the X-ray- and cryo-EM-based 3D reconstructions of other connexin channels feature a pocket corresponding to the antimalarial drug binding site described here, that is almost always filled by densities consistent with bound lipids or detergents (Supplementary Fig. S8). Structures of connexin 36 (Cx36) in a flexible NTH state (FN) and a pore-lining NTH (PLN) state have been elucidated by Lee et al [28]. Our analysis reveals a noteworthy similarity: the lipid-like density or NTH density observed in Lee et al.'s structures appears to bind within the same pocket where mefloquine binds in our structure (Supplementary Fig. S9). While this site in distinct connexin channels interacts non-specifically with hydrophobic small molecules, our structures show that this site is used by connexin-specific drugs.

Ion channel-targeted drug development is a major area of medicinal chemistry and pharmacology. Many of the drugs on the market or in phase II/III trials today target ligand- and voltage-gated ion channels, such as benzodiazepine diazepam [29], verapamil [30] and AXS-05 [31]. The importance of ion channels for drug discovery is also exemplified by the absolute requirement to test all newly developed drugs for cross-reactivity with HERG channel to prevent cardiotoxicity [32]. Connexin channels, including GJCs and HCs, also represent attractive drug targets and new therapies acting on Cx26 and Cx43 are currently under development [33]. Some of the known drugs with primary action unrelated to connexins (such as mefloquine, quinine and quinidine, the topic of this study) cross-react with Cx36. This reactivity may underlie the adverse effects of these drugs. The neurological and cardiac effects of mefloquine, quinine and quinidine may well correlate with the ability of these drugs to disrupt gap junction coupling in the brain or heart, respectively. Moreover, these effects may be mediated by Cx36 or by other connexin channels that might accommodate these drugs in the corresponding drug binding sites.

This mode of connexin inhibition by mefloquine has been confirmed in Cx32 and Cx43, combining the evidence from cryo-EM analysis, hemichannel and gap junction channel assays [34]. Therefore, the mode

of connexin channel inhibition by mefloquine, and by extension other drugs, such as quinine and quinidine, as presented here, may be conserved across the connexin channel family.

Our high-resolution structures can be leveraged to provide a foundational basis for the development of *in silico* drug discovery approaches. The following criteria would have to be satisfied to develop a connexin subtype-selective inhibitor: (i) extensive contacts and high complementary to the drug pocket; (ii) the presence of an asymmetric head-group mediating the contacts with the neighbouring drugs and with the residue equivalent to E43 in Cx36. These features could build on the existing geometry and the principles of antimalarial drug-mediated inhibition as described here, although other solutions may be found utilizing the same binding site but very different chemistry. Headgroup modification could potentially be used to fine-tune the permeability of the channels. This may be possible to accomplish due to the incomplete blockage of the channel by the drugs as in the case of mefloquine, evident from our structural and MD simulation data. Both the strength of drug-drug interactions in the connexin pore and the permeability of the drug-bound state of the channel to small solutes or ions may be possible to tailor to the specific connexin isoforms.

References

1 Zheng, S. Q. *et al.* MotionCor2: anisotropic correction of beam-induced motion for improved cryo-electron microscopy. *Nat Methods* **14**, 331-332 (2017). https://doi.org:10.1038/nmeth.4193

2 Zhang, K. Gctf: Real-time CTF determination and correction. *J Struct Biol* **193**, 1-12 (2016). https://doi.org:10.1016/j.jsb.2015.11.003

3 Scheres, S. H. RELION: implementation of a Bayesian approach to cryo-EM structure determination. *J Struct Biol* **180**, 519-530 (2012). https://doi.org:10.1016/j.jsb.2012.09.006

4 Zivanov, J. *et al.* New tools for automated high-resolution cryo-EM structure determination in RELION-3. *Elife* **7** (2018). https://doi.org:10.7554/eLife.42166

5 Kucukelbir, A., Sigworth, F. J. & Tagare, H. D. Quantifying the local resolution of cryo-EM density maps. *Nat Methods* **11**, 63-65 (2014). https://doi.org:10.1038/nmeth.2727

6 Emsley, P., Lohkamp, B., Scott, W. G. & Cowtan, K. Features and development of Coot. *Acta Crystallogr D Biol Crystallogr* **66**, 486-501 (2010). https://doi.org:10.1107/S0907444910007493

7 Adams, P. D. *et al.* PHENIX: a comprehensive Python-based system for macromolecular structure solution. *Acta Crystallogr D Biol Crystallogr* **66**, 213-221 (2010). https://doi.org:10.1107/S0907444909052925

8 Qi, C., Sorrentino, S., Medalia, O. & Korkhov, V. M. The structure of a membrane adenylyl cyclase bound to an activated stimulatory G protein. *Science* **364**, 389-394 (2019). https://doi.org:10.1126/science.aav0778

9 Chen, V. B. *et al.* MolProbity: all-atom structure validation for macromolecular crystallography. *Acta Crystallogr D Biol Crystallogr* **66**, 12-21 (2010). https://doi.org:10.1107/S0907444909042073

10 Schrodinger, L. The PyMOL molecular graphics system. *Version* **1**, 8 (2015).

11 Pettersen, E. F. *et al.* UCSF Chimera--a visualization system for exploratory research and analysis. *J Comput Chem* **25**, 1605-1612 (2004). https://doi.org:10.1002/jcc.20084

12 Pettersen, E. F. *et al.* UCSF ChimeraX: Structure visualization for researchers, educators, and developers. *Protein Sci* **30**, 70-82 (2021). https://doi.org:10.1002/pro.3943

13 Dolinsky, T. J., Nielsen, J. E., McCammon, J. A. & Baker, N. A. PDB2PQR: an automated pipeline for the setup of Poisson-Boltzmann electrostatics calculations. *Nucleic Acids Res* **32**, W665-667 (2004). https://doi.org:10.1093/nar/gkh381

14 Wang, J., Cieplak, P. & Kollman, P. A. How well does a restrained electrostatic potential (RESP) model perform in calculating conformational energies of organic and biological molecules? *Journal of computational chemistry* **21**, 1049-1074 (2000). https://doi.org:https://doi.org/10.1002/1096-987X(200009)21:12<1049::AID-JCC3>3.0.CO;2-F

15 Baker, N. A., Sept, D., Joseph, S., Holst, M. J. & McCammon, J. A. Electrostatics of nanosystems: application to microtubules and the ribosome. *Proc Natl Acad Sci U S A* **98**, 10037-10041 (2001). https://doi.org:10.1073/pnas.181342398

16 Perez-Riverol, Y. *et al.* The PRIDE database resources in 2022: a hub for mass spectrometry-based proteomics evidences. *Nucleic Acids Res* **50**, D543-D552 (2022). https://doi.org:10.1093/nar/gkab1038

17 Sousa da Silva, A. W. & Vranken, W. F. ACPYPE - AnteChamber PYthon Parser interfacE. *BMC Res Notes* **5**, 367 (2012). https://doi.org:10.1186/1756-0500-5-367

18 Jo, S., Kim, T., Iyer, V. G. & Im, W. CHARMM-GUI: a web-based graphical user interface for CHARMM. *J Comput Chem* **29**, 1859-1865 (2008). https://doi.org:10.1002/jcc.20945

19 Piana, S., Robustelli, P., Tan, D., Chen, S. & Shaw, D. E. Development of a Force Field for the Simulation of Single-Chain Proteins and Protein-Protein Complexes. *J Chem Theory Comput* **16**, 2494-2507 (2020). https://doi.org:10.1021/acs.jctc.9b00251

20 Abraham, M. J. *et al.* GROMACS: High performance molecular simulations through multi-level parallelism from laptops to supercomputers. *SoftwareX* **1**, 19-25 (2015).

21 Petersen, H. G. Accuracy and efficiency of the particle mesh Ewald method. *The Journal of chemical physics* **103**, 3668-3679 (1995).

22 Bernetti, M. & Bussi, G. Pressure control using stochastic cell rescaling. *J Chem Phys* **153**, 114107 (2020). https://doi.org:10.1063/5.0020514

23 Invernizzi, M. & Parrinello, M. Rethinking Metadynamics: From Bias Potentials to Probability Distributions. *J Phys Chem Lett* **11**, 2731-2736 (2020). https://doi.org:10.1021/acs.jpclett.0c00497

24 Rizzi, V., Aureli, S., Ansari, N. & Gervasio, F. L. OneOPES, a Combined Enhanced Sampling Method to Rule Them All. *Journal of Chemical Theory and Computation* **19**, 5731-5742 (2023). https://doi.org:10.1021/acs.jctc.3c00254

25 Ansari, N., Rizzi, V. & Parrinello, M. Water regulates the residence time of Benzamidine in Trypsin. *Nat Commun* **13**, 5438 (2022). https://doi.org:10.1038/s41467-022-33104-3

26 Aureli, S., Di Marino, D., Raniolo, S. & Limongelli, V. DDT - Drug Discovery Tool: a fast and intuitive graphics user interface for docking and molecular dynamics analysis. *Bioinformatics* **35**, 5328-5330 (2019). https://doi.org:10.1093/bioinformatics/btz543

27 C.P.van Zundert, G. & M.J.J. Bonvin, A. Fast and sensitive rigid-body fitting into cryo-EM density maps with PowerFit. *AIMS Biophysics* **2**, 73-87 (2015). https://doi.org:10.3934/biophy.2015.2.73

28 Lee, S. N. *et al.* Cryo-EM structures of human Cx36/GJD2 neuronal gap junction channel. *Nat Commun* **14**, 1347 (2023). https://doi.org:10.1038/s41467-023-37040-8

29 Dawson, G. R. *et al.* An inverse agonist selective for alpha5 subunit-containing GABAA receptors enhances cognition. *J Pharmacol Exp Ther* **316**, 1335-1345 (2006). https://doi.org:10.1124/jpet.105.092320

30 Pexton, T., Moeller-Bertram, T., Schilling, J. M. & Wallace, M. S. Targeting voltage-gated calcium channels for the treatment of neuropathic pain: a review of drug development. *Expert Opin Investig Drugs* **20**, 1277-1284 (2011). https://doi.org:10.1517/13543784.2011.600686

31 Tabuteau, H., Jones, A., Anderson, A., Jacobson, M. & Iosifescu, D. V. Effect of AXS-05 (Dextromethorphan-Bupropion) in Major Depressive Disorder: A Randomized Double-Blind Controlled Trial. *Am J Psychiatry* **179**, 490-499 (2022). https://doi.org:10.1176/appi.ajp.21080800

32 Sanguinetti, M. C. & Tristani-Firouzi, M. hERG potassium channels and cardiac arrhythmia. *Nature* **440**, 463-469 (2006). https://doi.org:10.1038/nature04710

33 Laird, D. W. & Lampe, P. D. Therapeutic strategies targeting connexins. *Nat Rev Drug Discov* **17**, 905-921 (2018). https://doi.org:10.1038/nrd.2018.138

34 Lavriha, P. *et al.* Mechanism of connexin channel inhibition by mefloquine and 2-aminoethoxydiphenyl borate. *bioRxiv* (2023). https://doi.org:10.1101/2023.12.11.571071

35 Laskowski, R. A. & Swindells, M. B. LigPlot+: multiple ligand-protein interaction diagrams for drug discovery. *J Chem Inf Model* **51**, 2778-2786 (2011). https://doi.org:10.1021/ci200227u

36 Thompson, J. D., Higgins, D. G. & Gibson, T. J. CLUSTAL W: improving the sensitivity of progressive multiple sequence alignment through sequence weighting, position-specific gap penalties and weight matrix choice. *Nucleic Acids Res* **22**, 4673-4680 (1994). https://doi.org:10.1093/nar/22.22.4673

37 Waterhouse, A. M., Procter, J. B., Martin, D. M., Clamp, M. & Barton, G. J. Jalview Version 2--a multiple sequence alignment editor and analysis workbench. *Bioinformatics* **25**, 1189-1191 (2009). https://doi.org:10.1093/bioinformatics/btp033

38 Maeda, S. *et al.* Structure of the connexin 26 gap junction channel at 3.5 A resolution. *Nature* **458**, 597-602 (2009). https://doi.org:10.1038/nature07869

39 Flores, J. A. *et al.* Connexin-46/50 in a dynamic lipid environment resolved by CryoEM at 1.9 A. *Nat Commun* **11**, 4331 (2020). https://doi.org:10.1038/s41467-020-18120-5

40 Qi, C. *et al.* Structures of wild-type and selected CMT1X mutant connexin 32 gap junction channels and hemichannels. *Sci Adv* **9**, eadh4890 (2023). https://doi.org:10.1126/sciadv.adh4890

41 Qi, C. *et al.* Structure of the connexin-43 gap junction channel in a putative closed state. *Elife* **12** (2023). https://doi.org:10.7554/eLife.87616

42 Brotherton, D. H., Savva, C. G., Ragan, T. J., Dale, N. & Cameron, A. D. Conformational changes and CO(2)-induced channel gating in connexin26. *Structure* **30**, 697-706 e694 (2022). https://doi.org:10.1016/j.str.2022.02.010

Supplementary Figures

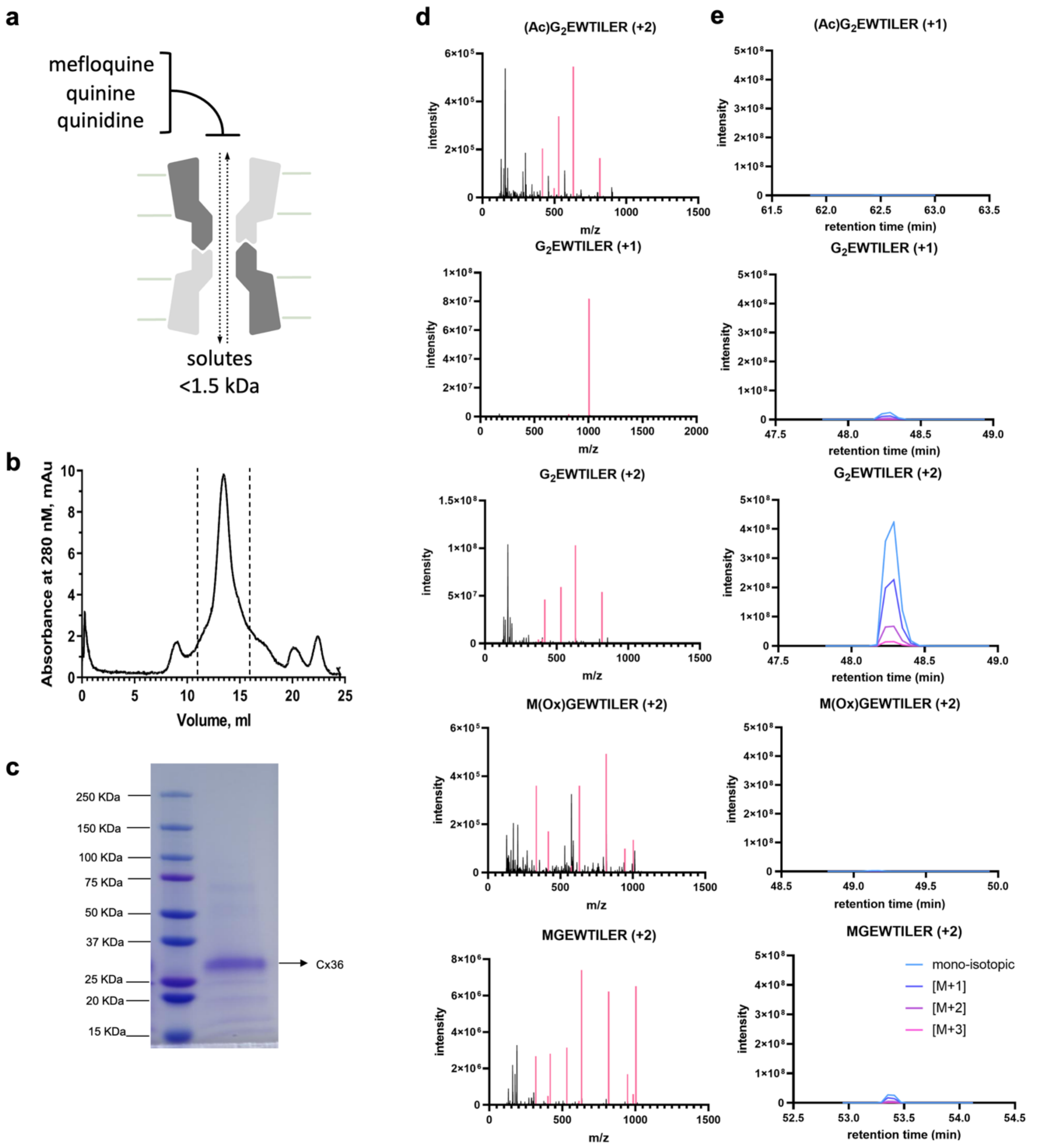

Supplementary Fig. S1. Expression, purification and mass spectrometric characterization of Cx36. a, A sketch illustrating the Cx36-mediated solute translocation and inhibition by selected small molecules. **b,** Size exclusion chromatogram (SEC) of Cx36 purification. **c,** SDS-PAGE of Cx36 purification. **d-e,** Mass spectrometric characterization of Cx36. Fragment spectra for each of the identified peptides **(d)**. Extracted-ion chromatograms (XIC) for fragment and parent ions detected in MS2 and MS1 spectra **(e)**.

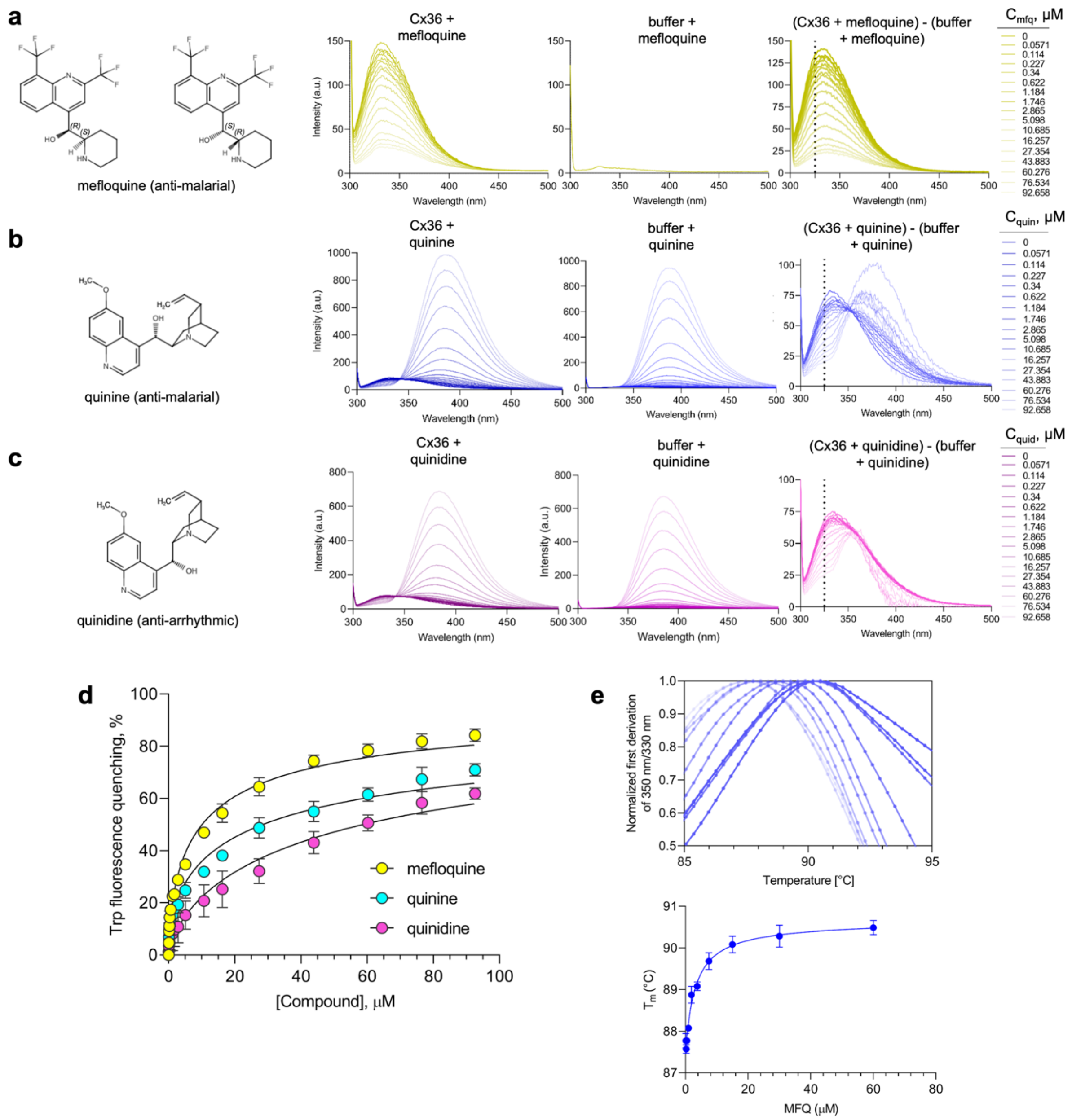

Supplementary Fig. S2. Tryptophan fluorescence quenching of Cx36 in response to treatment with different drugs: mefloquine (a), quinine (b), quinidine (c). The left panels display the fluorescence of Cx36 in the presence of varying concentrations of each drug, while the middle panels show the fluorescence in buffer containing different concentrations of the respective drug. In the case of mefloquine, the middle panel exclusively displays the fluorescence in the presence of 100 μM mefloquine within the buffer (a). The right panels illustrate the subtraction of these two fluorescence signals. Dot lines indicate the wavelength used for plotting the binding curves. Data is represented as mean (n=3). **d,** Tryptophan fluorescence quenching-based binding assays of three drugs with purified Cx36 (using data from a-c); the apparent K_d values are 10.4 ± 1.3 μM (mefloquine), 29 ± 5.3 (quinine), 58 ± 9 (quinidine); the values are expressed as mean ± S.D. (n = 3); the K_d mean values are significantly different from each other, judged by one way ANOVA. **e,** Thermal unfolding (nano-DSF) graphs of Cx36 upon treatment with mefloquine. Thermal unfolding data is represented as normalization of the first derivative of the ratio of fluorescence intensity at 330 nm and 350 nm. The unfolding transition temperature (Tm), the temperature at which 50% of the protein is unfolded, is plotted against the

concentration of mefloquine and fitted with a one-site binding model using GraphPad Prism software. Data is represented as mean ± SEM (n=3).

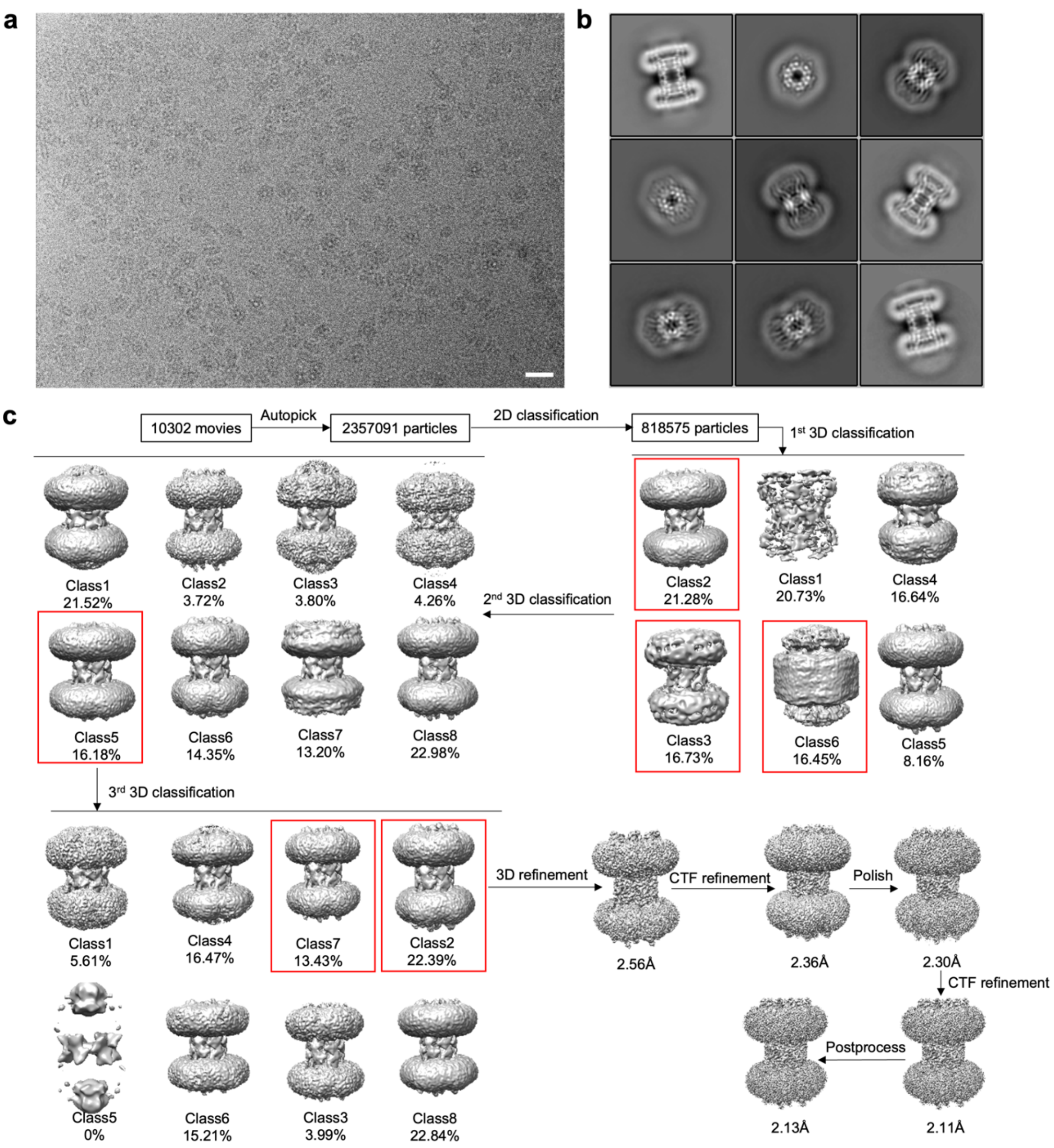

Supplementary Fig. S3. Cryo-EM data processing pipeline of Cx36-mfq in GDN. a, A representative micrograph of Cx36-mfq sample; the scale bar corresponds to 20 nm. **b,** representative 2D classes. **c,** Cryo-EM data process scheme for 3D reconstitution of Cx36-mfq in GDN.

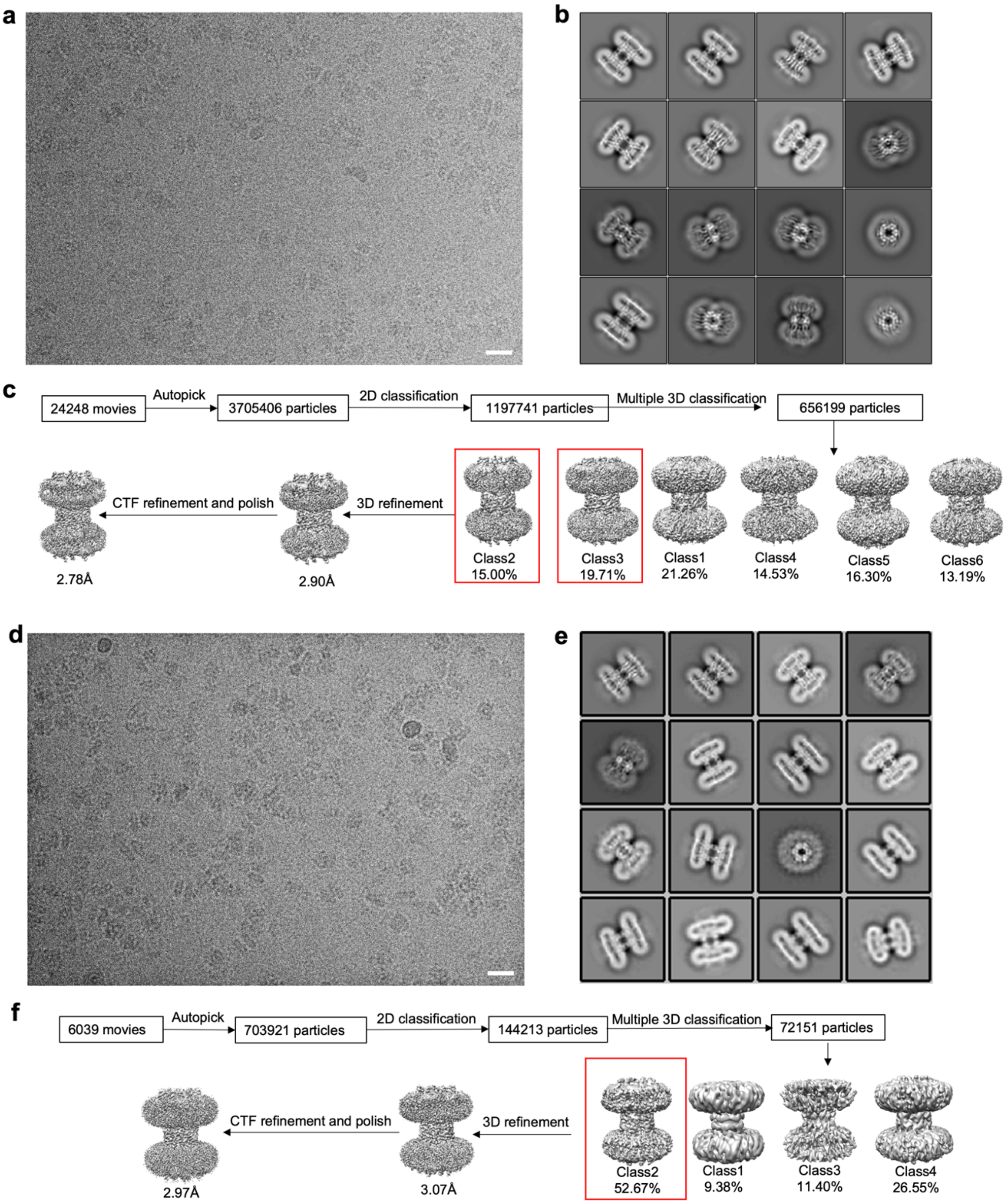

Supplementary Fig. S4. Cryo-EM data processing pipeline of Cx36-quin (a-c) and Cx36-quid (d-f) in GDN. a, A representative micrograph of Cx36-quin sample; the scale bar corresponds to 20 nm. **b,** representative 2D classes. **c,** Cryo-EM data process scheme for 3D reconstitution of Cx36-quin in GDN. **d,** A representative micrograph of Cx36-quid sample; the scale bar corresponds to 20 nm. **e,** representative 2D classes. **f,** Cryo-EM data process scheme for 3D reconstitution of Cx36-quid in GDN.

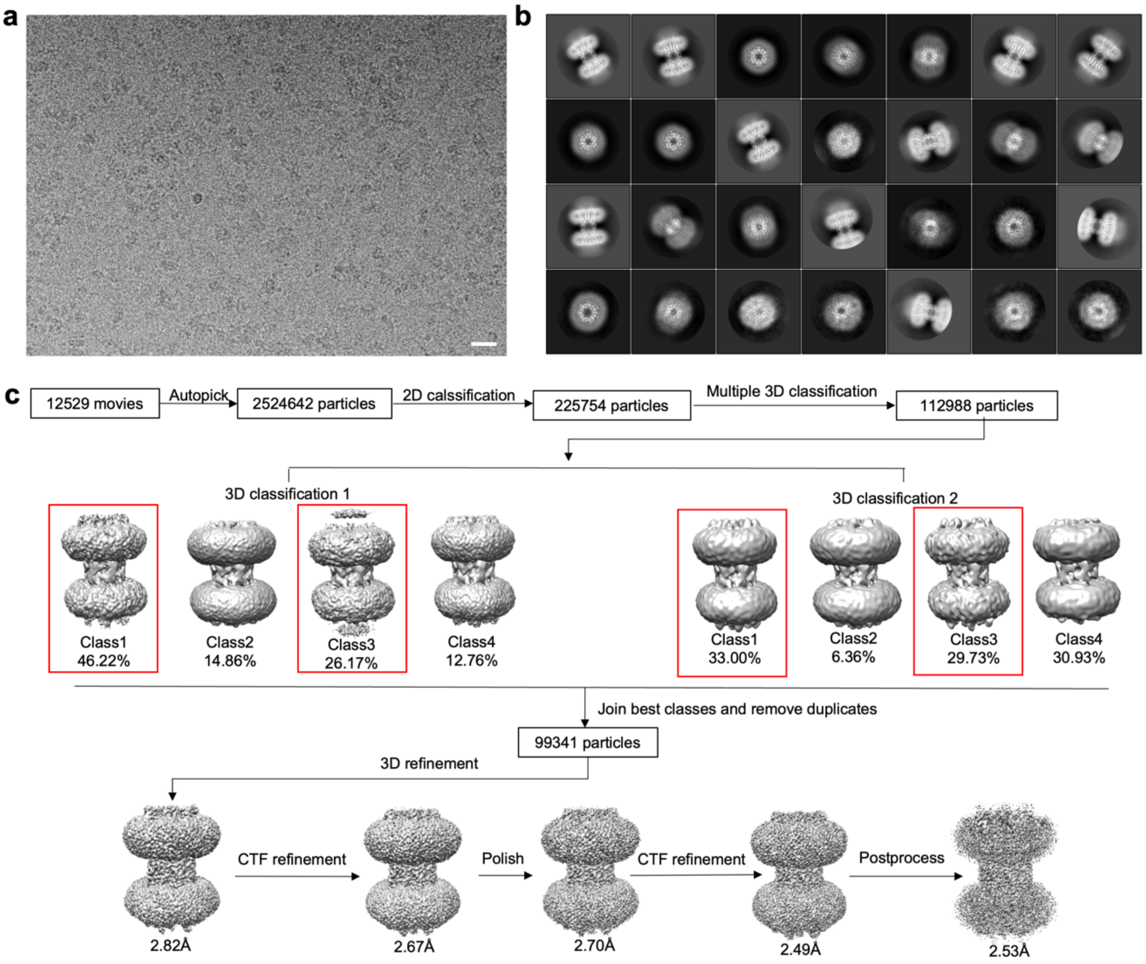

Supplementary Fig. S5. Cryo-EM data processing pipeline of apo-Cx36 in GDN. a, A representative micrograph of apo-Cx36 sample; the scale bar corresponds to 20 nm. **b,** representative 2D classes. **c,** Cryo-EM data process scheme for 3D reconstitution of Cx36 in GDN.

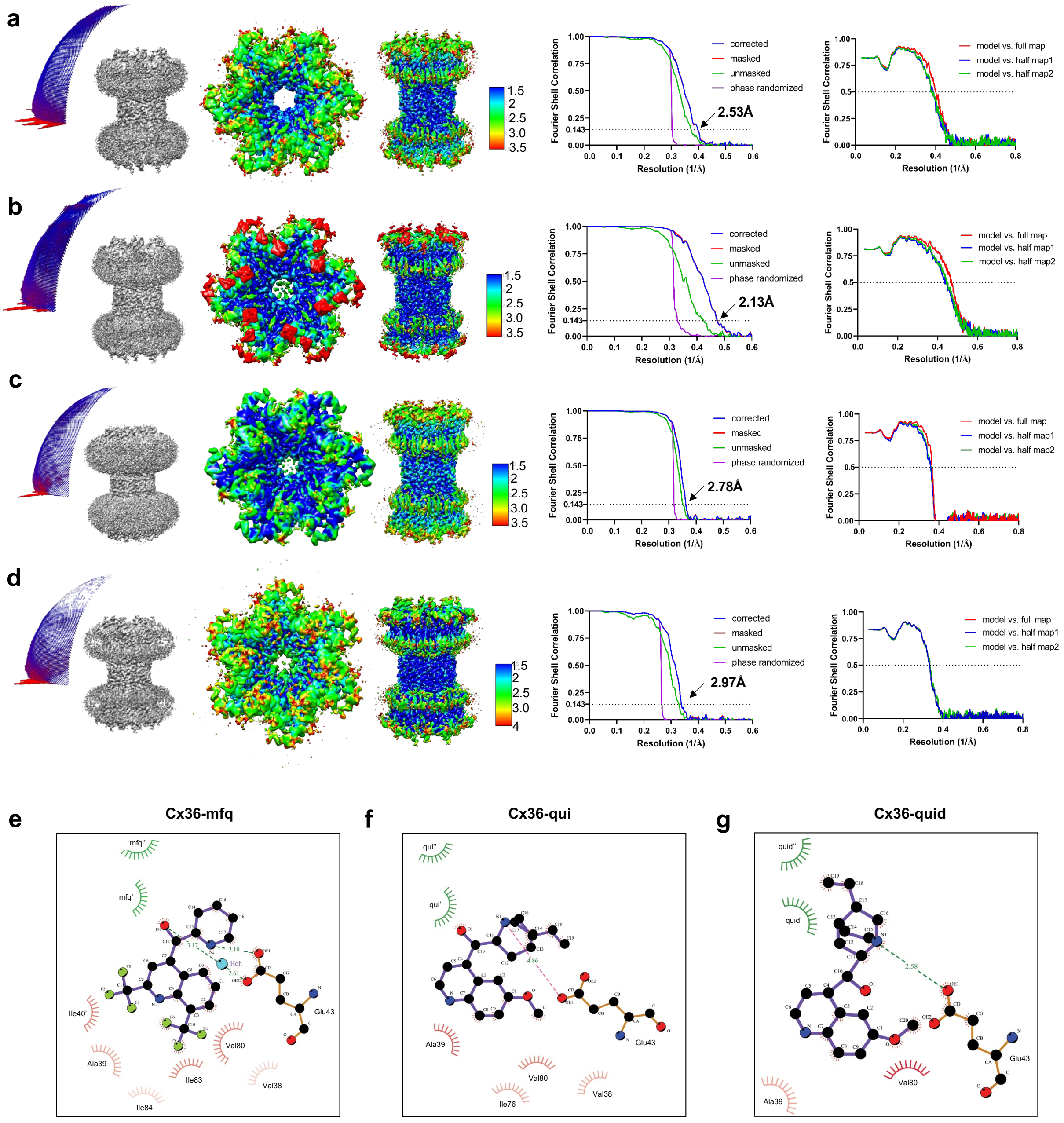

Supplementary Fig. S6. Angular distribution, local resolution maps and Fourier shell correlation (FSC) plots of Cx36 with and without drugs bound, and 2D plots of the ligand binding sites. a, apo-Cx36. **b,** Cx36-mfq. **c,** Cx36-quin. **d,** Cx36-quid. **e-h,** 2D Plots of the three ligand binding pockets: mefloquine (e), quinine (f) and quinidine (g), generated using LigPlot+ [35]. Hydrogen bonds are represented by green dotted lines. Hydrophobic interactions between the ligands and residues within 2.9 Å to 3.9 Å are depicted in red. The transparency of the red color increases with longer distances, indicating a higher level of transparency for interactions with greater distances.

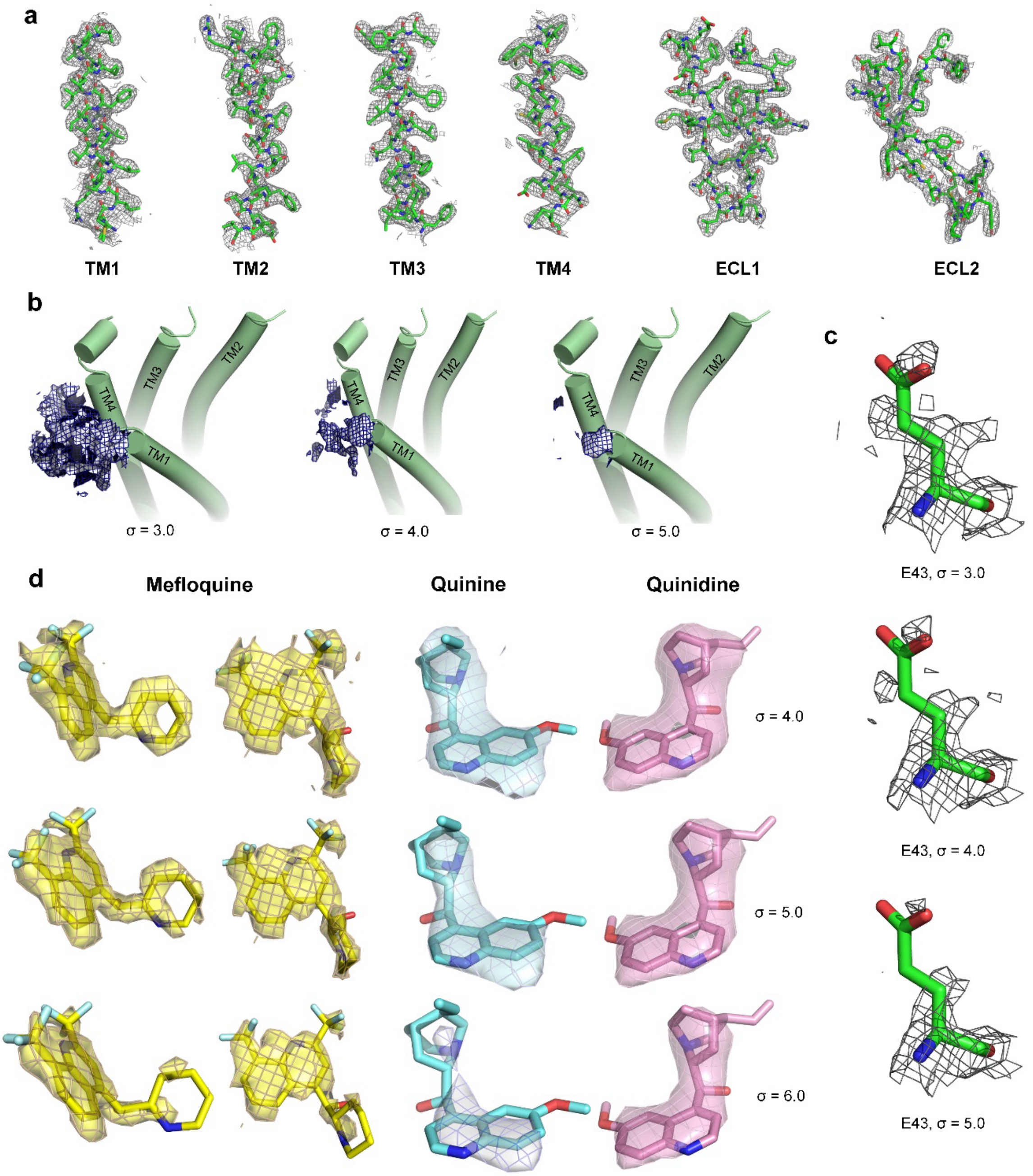

Supplementary Fig. S7. Cryo-EM density map features of Cx36 and drugs. a, Representative cryo-EM density map features of Cx36 gap junction channel (GJC) with mefloquine, including the transmembrane (TM) helixes and extracellular loop 1 (ECL1) and extracellular loop 2 (ECL2). **b,** Cryo-EM density map features of N-terminal at σ levels 3.0, 4.0, 5.0. **c,** Cryo-EM density map features of Glu43 in the Cx36-mfq map. **d,** Cryo-EM density map features of mefloquine (viewed at two angles, illustrating the well-resolved densities corresponding to the rings), quinine and quinidine (viewed at one angle, showing less well-resolved head-group ring) at σ levels 4.0, 5.0, 6.0 each.

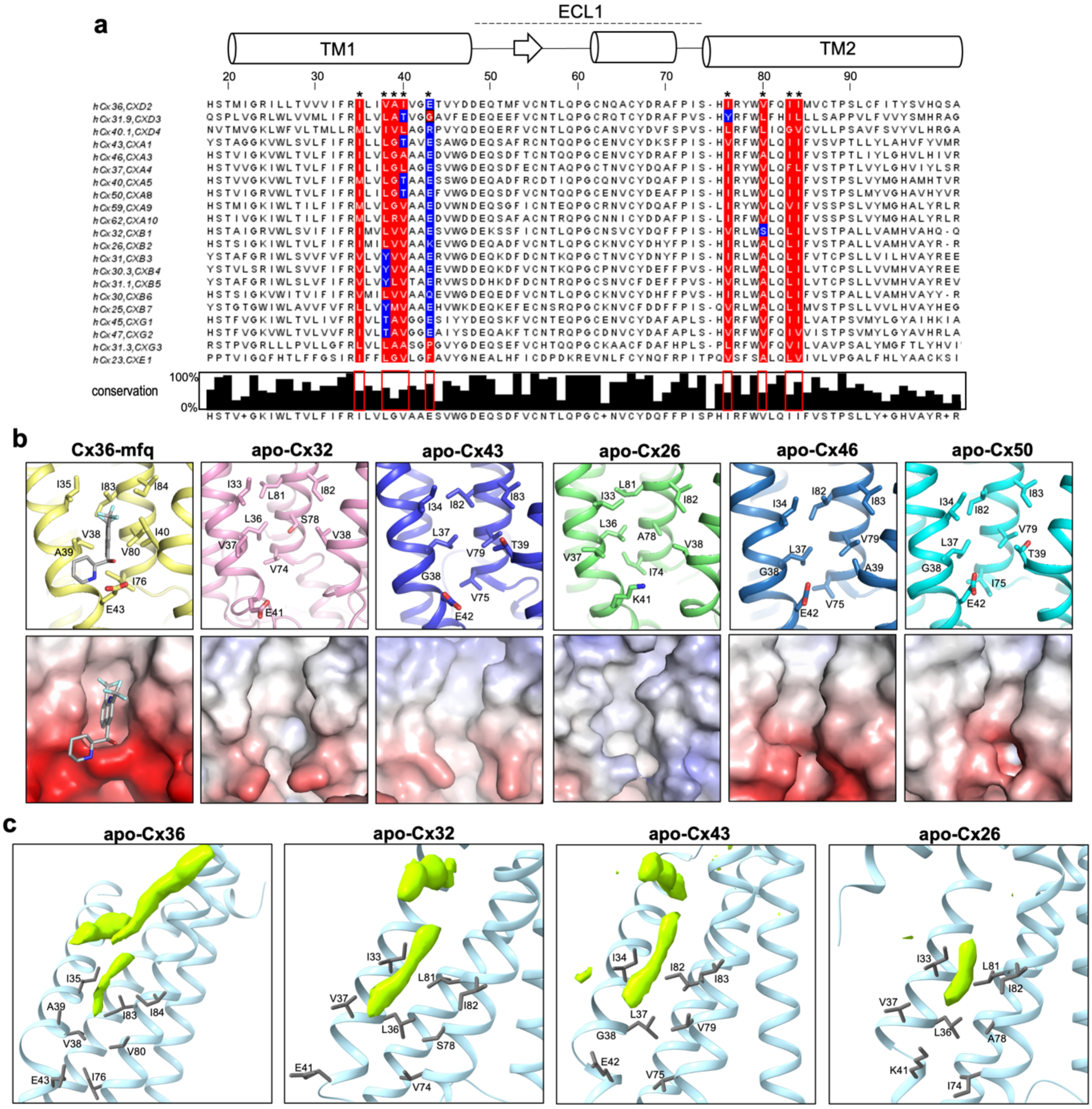

Supplementary Fig. S8. Amino acid sequence alignment and structural comparison in connexin families. a, Amino acid sequence alignment of all human connexins in the mefloquine binding site. Sequences are aligned with CLUSTALW [36]. Secondary structures and numbering of residues of Cx36 are represented at the top. The hydrophobic and hydrophilic residues are shaded in red and blue, respectively. Asterisks indicate ligand-binding residues in Cx36. The figure is created with Jalview [37] and manually modified. **b,** Structural comparison of connexins in the mefloquine binding site. Mefloquine-bound structures of Cx36, Cx32 (PDB 7ZXM) [34], Cx43 (PDB 7Z22) [34] and nonligand-bound structures of Cx26 (PDB 2ZW3, 3.50 Å) [38], Cx46 (PDB 7JKC) [39], Cx50 (PDB 7JJP) [39] are used. Each panel below shows the electrostatic surface potential representation of binding pocket. **c,** Densities of lipids shown in green are present in the ligand binding sites of Cx36, Cx32 (EMD 15010) [40], Cx43 (EMD 14455) [41], Cx26 (EMD 13938, 2.20 Å) [42].

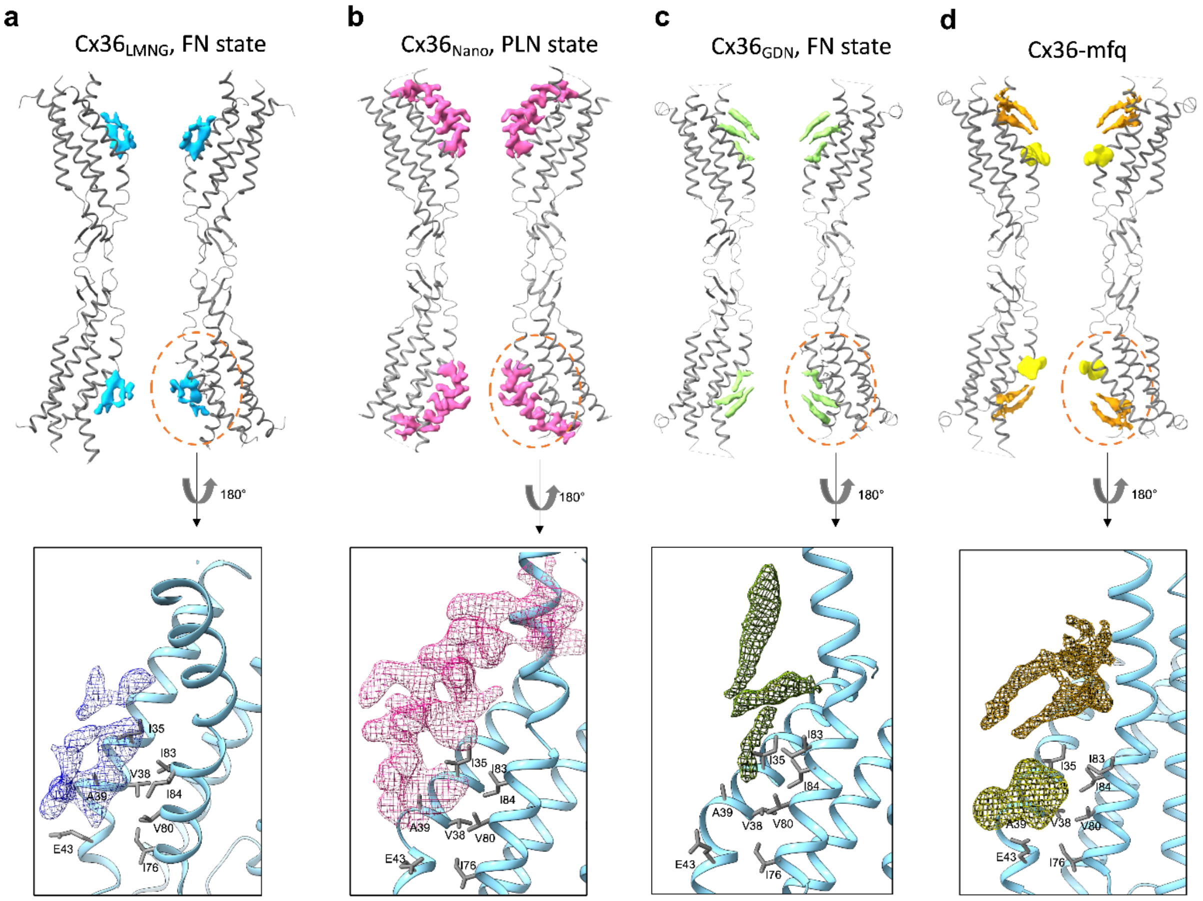

Supplementary Fig. S9. Comparison of densities in the hydrophobic binding pocket of Cx36. a, Structure of Cx36 in LMNG-CHS (PDB 7XKK, EMD 33256) [28] in free N-terminal helices state (FN). Lipid-like density is shown in blue. **b,** Structure of Cx36 in nanodisc (PDB 7XNH, EMD 33315) [28] in pore-lining N-terminal helices state (PLN). NTH density is shown in magenta. **c,** Structure of Cx36 in GDN-CHS (PDB 8R7P, EMD 18987) in FN state, described here. Lipid-like density is shown in green. **d,** Structure of Cx36-mfq in GDN-CHS (PDB 8QOJ, EMD 18540), described here. The density of mefloquine is shown in yellow and Lipid-like density is shown in orange.

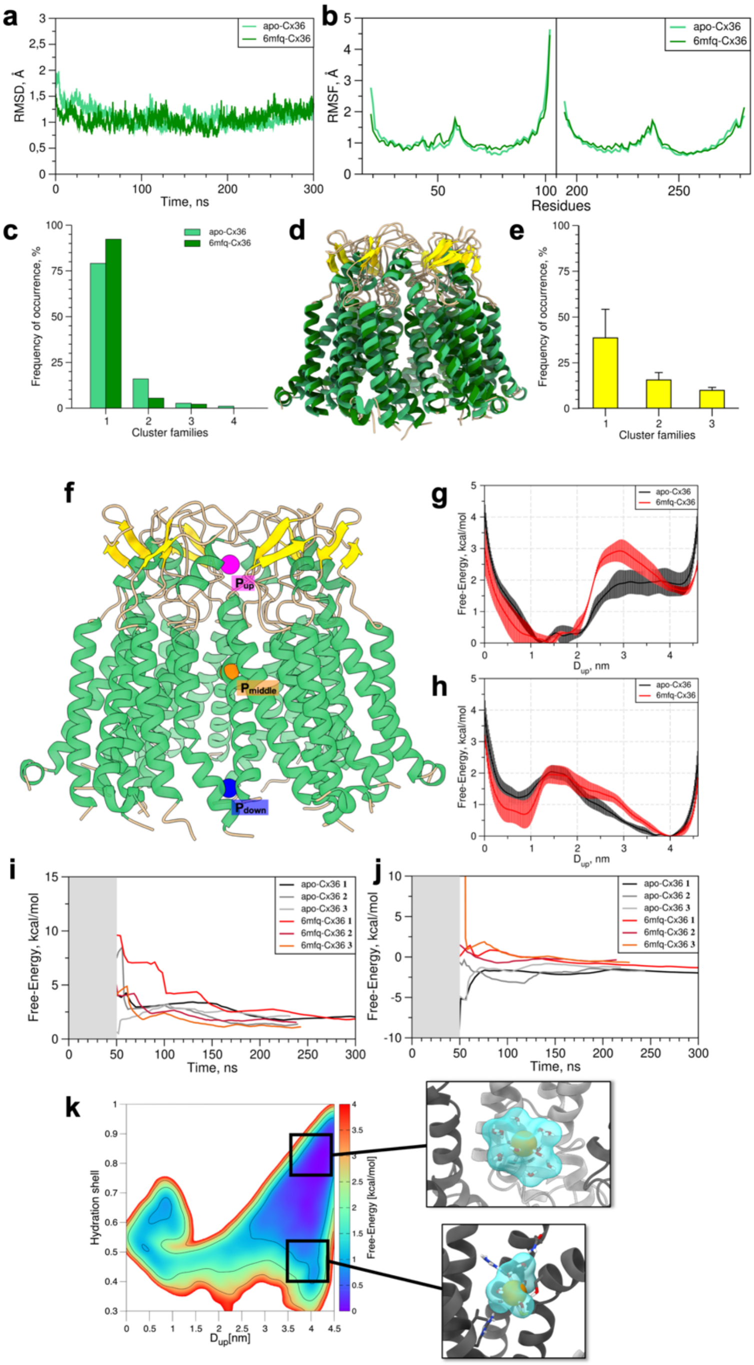

Supplementary Fig. S10. MD simulations of the *apo-Cx36* and *6mfq-Cx36* systems. a, A plot reporting the average RMSD of the secondary structure Cα atoms of Cx36 hexamer; **b,** A plot reporting the average per-residue RMSF of each Cx36 monomer. The left panel covers the residues from S19 to A102, whereas the right panel covers the residues from G194 to L282; **c,** Histograms displaying the frequency of occurrence of each cluster family in the *apo-Cx36* and *6mfq-Cx36* MD simulations; **d,** Superimposition of the centroids of the most relevant cluster families measured for the *apo-Cx36* and *6mfq-Cx36* simulations. For *apo-Cx36*, α-helices are displayed as green ribbons and the ß-strands as yellow arrows. For *6mfq-Cx36*, α-helices are displayed as dark green ribbons and the ß-strands as gold arrows; **e,** Histogram displaying the frequency of occurrence of mefloquine's cluster families in the *6mfq-Cx36* MD simulation. **f-j,** Details of the OPES simulations carried out on the *apo-Cx36* and *6mfq-Cx36* systems. **f,** A schematic depiction of a set of dummy atoms employed to carry out the OPES simulations. The dummy atoms P_{up}, P_{middle}, and P_{down} are represented as spheres and colored in magenta,

orange, and blue, respectively. The α-helices and ß-strands of Cx36 are colored in green and yellow, respectively. **g-h,** Free-energy profiles of the translation of K^+ (**g**) and Cl^- (**h**) along Cx36's pore in the *apo-Cx36* and *6mfq-Cx36* OPES simulations. Each OPES simulation was repeated three times. The error bars represent the standard deviation among the 3 replicas. **i-j,** ΔG of translation as function of the simulation time for the K^+ (**i**) and Cl^- (**j**) ions in the OPES simulations performed on the *apo-Cx36* and *6mfq-Cx36* systems. The ΔG of translation estimated for the *apo-Cx36* systems are colored in shades of grey, whereas the ΔG of translation for the *6mfq-Cx36* systems are colored in shades of red. **k,** Free-energy surfaces associated with Cl- permeation across the Cx36 hexamer in the *apo-Cx36*. The insets show two representative frames of the different hydration state of Cl- ion in the intracellular portion of the Cx36 hexamer.

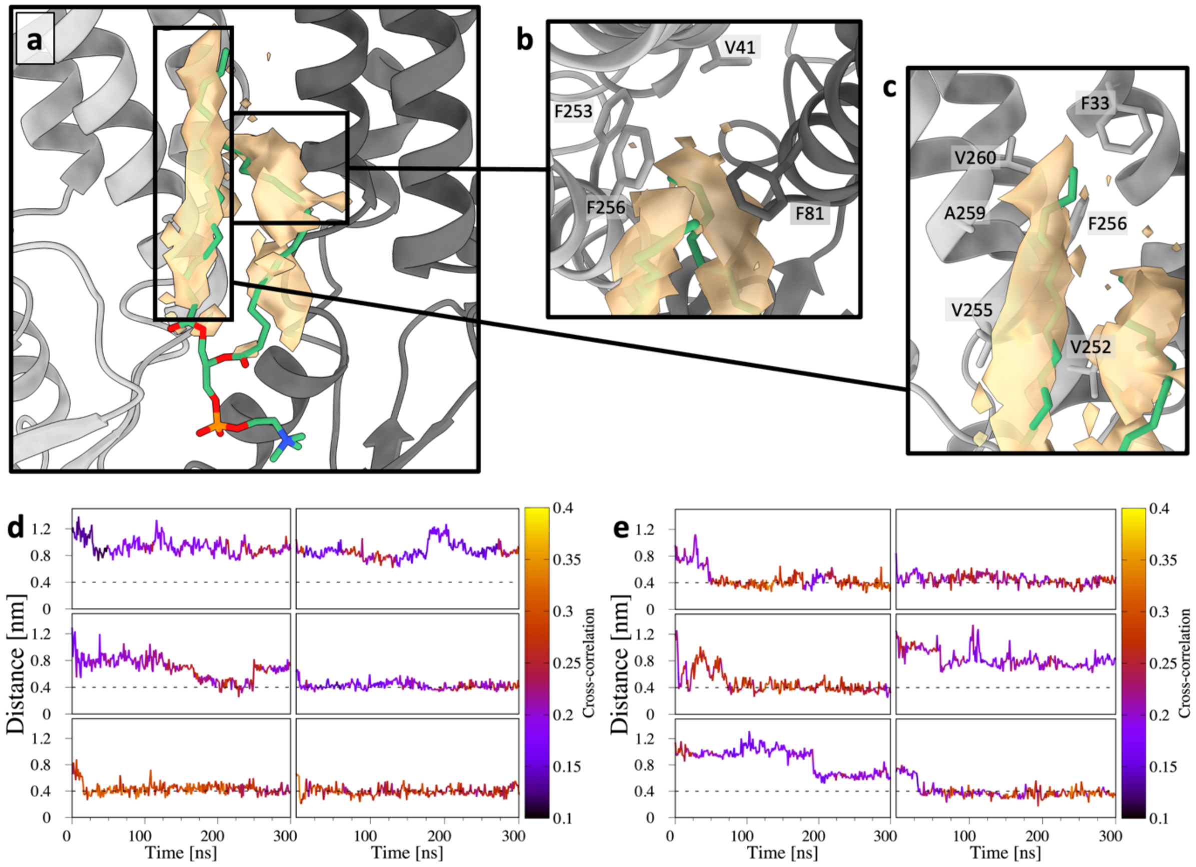

Supplementary Fig. S11. Phospholipid interactions at the protein-lipid interface during the MD simulations. a, Fitting of a POPC molecule into two lipid-like cryo-EM densities located at the interface between two Cx36 monomers. **b,** Placement of POPC's oleic acid chain within the hydrophobic hotspot, overlaid with the first lipid-like density. **c,** Binding mode of POPC's palmitic acid chain onto the P247-L275 helix of Cx36, aligned with the second lipid-like density. The two adjacent Cx36 monomers are colored in dark and light grey, the POPC molecule is colored in light green, and the lipid-like density are colored in ochre. **d-e,** Distances of six POPC oleic acid chains relative to the core of the hydrophobic hotspots during the *apo-Cx36* (**d**) and *6mfq-Cx36* (**e**) MD simulations. Cross-correlation with the Cryo-EM map is indicated by the color bar on the right side and is measured through the rigid-body fitting software "Powerfit" [27].

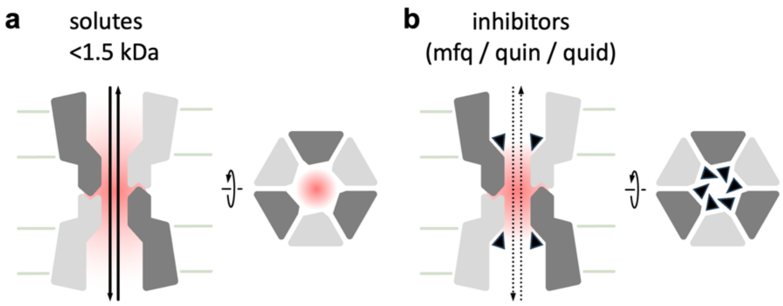

Supplementary Fig. S12. A structure-based model of drug-mediated Cx36 inhibition. a, Under basal conditions, Cx36 (as other connexin GJCs) is permeable to solutes with a molecular weight cut-off in the ~1-1.5 kDa range. The electrostatic properties of the pore enable solute movement. **b,** Upon drug binding as observed in our cryo-EM 3D reconstructions (mefloquine - “mfq”, quinine - “quin”, quinidine - “quid”, shown as black triangles), steric obstruction is introduced into the path of the solutes and the electrostatic properties of the pore are altered, resulting in reduced solute flux (dotted arrows).

Supplementary Tables

Supplementary Table S1. Cryo-EM analysis and statistics

**Data collection**				
Sample	apo-Cx36	Cx36-mfq	Cx36-quin	Cx36-quid
Instrument	FEI Titan Krios / Gatan K3 Summit			
Voltage (kV)	300			
Electron Dose (e-/Å2)	50	55	50	55
Defocus range (μm)	-0.5 to -2.5			
Pixel size (Å)	0.65	0.66	0.66	0.66
Number of particles	99341	102585	231305	39915
FSC threshold 0.143	2.53	2.13	2.78	2.97
**Refinement**				
Model resolution FSC threshold 0.5	2.49	2.14	2.73	2.90
Map sharpening B-factor (Å)	-84.6697	-51.3034	-103.162	-90.2861
Map CC	0.96	0.94	0.81	0.84
**Model composition**				
Protein residues/ligands/water	2076/0/90	2076/12/162	2076/12/0	2076/12/0
Bond length r.m.s.d (Å)	0.003	0.002	0.004	0.003
Bond angle r.m.s.d (°)	0.461	0.508	0.574	0.508
**Validation**				
MolProbity score	1.07	1.17	1.31	1.27
Clash score	2.82	2.43	3.68	3.57
Rotamer outlier (%)	0.53	1.55	1.60	1.44
Ramachandran plot				
Favoured (%)	98.62	99.31	99.41	99.31
Allowed (%)	1.38	0.69	0.59	0.69
Disallowed (%)	0.00	0.00	0.00	0.00

Supplementary Table S2. Crossing events of the K^+ and Cl^- ions in the classical MD simulations carried out on the *apo-Cx36* and *6mfq-Cx36* systems.

	**apo-Cx36**	**6mfq-Cx36**
**K^+ ions**	30	7
**Cl^- ions**	6	2
